# *Rothia nasimurium* as a Cause of Disease: First Isolation from Farmed Geese

**DOI:** 10.3390/vetsci9050197

**Published:** 2022-04-21

**Authors:** Yuhui Kang, Hongshan Zhou, Wenjie Jin

**Affiliations:** 1 College of Veterinary Medicine, Yangzhou University, Yangzhou 225009, China; vet-kyhh@outlook.com (Y.K.); hongshanzhou@outlook.com (H.Z.); 2 Jiangsu Co-Innovation Center for Prevention and Control of Important Animal Infectious Diseases and Zoonosis, Yangzhou 225009, China; 3 Ministry of Education Key Laboratory of Poultry Preventive Medicine, Yangzhou 225009, China

**Keywords:** *Rothia nasimurium*, goose, depilation, multidrug resistance

## Abstract

*Rothia nasimurium* was known previously as an opportunistic pathogen of animals. However, there are few reports regarding the pathogenicity of *Rothia nasimurium*. In September 2020, geese contracted a disease of unknown cause which brought economic losses to a farm in Jiangsu Province, China, prompting a series of investigations. The bacterium was isolated, cultured, and purified, and then identified using Gram staining, biochemical tests, matrix-assisted laser desorption/ionization time of flight mass spectrometry, and 16S rRNA sequence analysis. After determining the obtained bacteria species, antibiotic susceptibility tests and animal regression experiments were carried out. A strain of bacterium was successfully isolated from the livers of the diseased geese, which was identified as a strain of the Gram-positive bacterium *Rothia nasimurium* according to the 16S rRNA sequencing results. By indexing references, no goose was reported to have been infected with *Rothia nasimurium.* The antibiotic susceptibility testing showed that only four antibiotics (amikacin, cefazolin, fosfomycin, and ampicillin/sulbactam) could effectively inhibit the growth of the *Rothia nasimurium* strain. The animal regression experiments showed that the novel isolated strain could infect goslings, and it also causes serious depilation of goslings. The results of the manuscript expanded the range of pathogenic microorganisms in geese, which is helpful to develop methods for avian endemic control.

## 1. Introduction

In September 2020, several 8-month-old geese showed inactivity, crouching, and unsteady gait with unknown cause and no relief after taking azithromycin and levofloxacin, bringing economic losses to a farm in Jiangsu Province, China. Two diseased geese were randomly selected for diagnosis, and bacteria were isolated from the liver. The bacteria isolated were identified as *Rothia nasimurium*, which have not been reported in geese before. *Rothia nasimurium*, from the family Micrococcaceae, was isolated and named by Collins et al. in 2000 [1]. It is a Gram-positive facultative anaerobic cocci. The bacterium was isolated from the nose of a healthy mouse; therefore, it was named *Rothia nasimurium*. Members of the genus *Rothia* are commonly regarded as part of the normal flora of the oral and intestinal tract of humans, pigs, and rodents [2,3,4]. In 2011, Li isolated a strain of *Rothia nasimurium* (No. 91) from the air of a farm, which presented high-level resistance to all tested antibiotics except vancomycin and had never been seen among all airborne bacteria and had not been reported in *Rothia* previously [5]. In 2014, Bemis et al. [6] isolated a strain from a dog’s skin lesions, tonsils, external ear canal, and semen, which caused strong synergistic hemolysis with *Staphylococcus* colonies in primary culture. This indicated that *Rothia nasimurium* is an opportunistic pathogen. In 2015, Hansen et al. isolated *Rothia nasimurium* in anser albifrons’s eggs, which was the first time that *Rothia* species were reported to have been found in bird eggs [7]. In 2017, another strain of *Rothia nasimurium* was isolated from the tonsils of healthy piglets by Gaiser et al., who found that the isolate could inhibit the growth of multiple strains and serotypes of the porcine pathogen *Streptococcus suis*. That was the first report of a normal bacterium from mammal-associated microbiota that contained a nonribosomal peptide synthetase (NRPS) gene cluster encoding a valinomycin-type nonribosomal peptide [8]. In 2021, Wang et al. isolated the E1706032a strain of *Rothia nasimurium*, which displayed wide antibiotic resistance and dissemination potential via mobile elements and was claimed to have the potential to cause difficult-to-treat infections in animals and humans [9]. In the same year, Zhao et al. isolated a strain of *Rothia nasimurium* from a rabbit and reported that it could cause disease [10]. However, little attention has been paid to *Rothia nasimurium,* which is essential for further study.

In this study, a strain of *Rothia nasimurium* was isolated from diseased geese, which suggested that *Rothia nasimurium* is able to infect geese and make them ill. Consequently, we carried out a series of studies to further explore the characteristics of this bacterium, with the aim of reducing its potential harm to the geese.

## 2. Materials and Methods

### 2.1. Isolation and Purification

Two 8-month-old diseased geese showing inactivity, crouching, and unsteady gait were dissected using standard laboratory methods [11]. On a clean bench, the livers of the diseased geese were isolated aseptically, and then the bacteria from the depths of the livers were swabbed and inoculated onto blood agar medium (Bkman, Changsha, China) and cultured in a 37 °C incubator for 12 h [12,13,14], after which a single colony could be selected. The single colony was inoculated onto a new blood agar plate and crossed in three zones for further purification. After growth overnight at 37 °C [15,16], a purified bacterial colony was eventually obtained.

### 2.2. Bacterial Identification

#### 2.2.1. Observation of Gram Staining

The pure cultured single colonies were coated on glass slides for Gram staining [17] and observed under an oil immersion microscope.

#### 2.2.2. Bacterial Biochemical Test

The purified single bacterial colony was cultured in Luria-Bertani liquid medium overnight, and then inoculated into micro biochemical reaction tubes purchased from Binhe Microorganism Reagents (Hangzhou, China), in which the bacteria were grown using glucose, lactose, maltose, mannitol, and sucrose, separately, as the sole carbon sources, and then placed in 37 °C incubator for 24 h. The bacteria were also inoculated into sodium citrate and urea and incubated at 37 °C for 72 h. The biochemical characteristics of the isolated bacteria were preliminarily identified by observing their growth in the above media.

#### 2.2.3. Matrix-Assisted Laser Desorption/Ionization Time-of-Flight Mass Spectrometry (MALDI-TOF MS)

Bacterial identification was performed using a Bruker mass spectrometer (Billerica, MA, USA) and calibrated using the Brucker bacterial test standard. The Brucker MBT_FC. par method was selected to collect the data automatically [18,19]. The score standards for mass spectrometry identification were as follows: 2.300–3.000 for completely reliable identification at the species level; 2.00–1.99 for identification at the species level; 1.700–1.999 for identification at the genus level; 0.000–1.699 indicated no credible identification.

### 2.3. Sequence Analysis of 16S rRNA

Four purified single colonies were selected randomly from the blood agar plate, and the bacterial DNA was extracted using a TIANamp Bacteria DNA kit (Tiangen Biotech, Beijing, China). The DNA concentration was measured using an enzyme labeling instrument. The extracted 16S rRNA was amplified using a Bacterial 16S rRNA amplification kit (Takara Bio, Dalian, China). The PCR reaction was carried out in an automated thermal cycler using the following cycling profile: initial denaturation of 94 °C for 5 min; 30 cycles of denaturation, annealing, and extension at 94 °C for 1 min, 55 °C for 1 min, and 72 °C for 1.5 min; a final extension period at 72 °C for 5 min. The PCR products were stained with ethidium bromide and loading buffer (Takara Bio, Dalian, China) and then subjected to electrophoresis through 1% agarose gels, purified, and sequenced. Analysis of the sequence data allowed bacterial identification. Then, the DNA sequences of the isolated strain were aligned with the NCBI database and generated a phylogenetic tree using MEGA7.

### 2.4. Antibiotic Susceptibility Testing

The K-B drug sensitive disk diffusion method was used [20,21], in which 50 μL of pure cultured bacterial solution (0.5 McFarland (MCF) units) was spread on a Mueller–Hinton agar plate. The Mueller–Hinton Broth was purchased from Hope Bio-Technology (Qingdao, China). Then, 20 kinds of common antibiotic disks purchased from Binhe Microorganism Reagents were placed on the surface of the plate uniformly. The diameter of the bacteriostatic rings was observed and measured after culturing at 37 °C for 24 h. According to the standards of the Clinical and Laboratory Standards Institute (CLSI), the drug sensitivity of the isolated strain was evaluated [22,23].

### 2.5. Animal Regression Experiment

A total of 15 1-day-old healthy goslings were bought from Jiali Goose Farm and numbered from 1 to 15. A single bacterial colony was cultured overnight in Luria–Bertani liquid medium until it reached 0.5 MCF. Goslings encoded with 1–3 were injected intraperitoneally (IP) with 0.2 mL of the culture containing 5 × 10^7^ cfu; goslings encoded with 4–5 were IP with 0.2 mL of the culture containing 5 × 10^5^ cfu; goslings encoded with 6–8 were given 0.2 mL of the culture containing 5 × 10^7^ cfu orally; goslings encoded with 9–10 were given 0.2 mL of the culture containing 5 × 10^5^ cfu orally; goslings encoded with 11–15 were used as negative controls. The infected goslings and the negative goslings were kept separately under the same breeding conditions, with plenty of clean water and food. When the geese showed abnormalities, their livers were removed and Swiss staining and bacterial isolation were performed. At the end of the experiment, all goslings were administered with pentobarbital sodium (150 mg/kg, IP) and sent to the Experimental Animal Incineration Center after confirmed death. This study was approved by the Institutional Review Board of Yangzhou University (Approval number: SYXK(SU)2021-0027). All methods were carried out in accordance with relevant guidelines and regulations, and the study was carried out in compliance with the ARRIVE guidelines.

## 3. Results

### 3.1. Isolation and Purification

**Figure 1:**
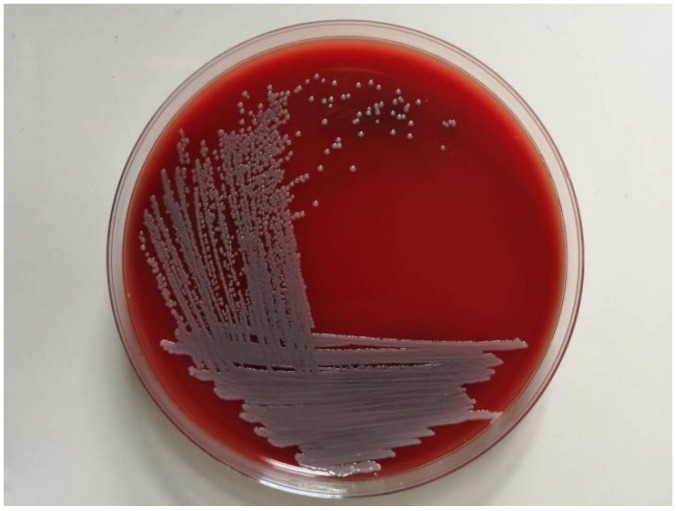
Purified bacterial colonies cultured on blood agar.

All geese in the farm were fully immunized with the usual vaccines. The farm adopts a free-range farming mode (rearing on floor), where geese could move around to obtain food and water freely. The farm was cleaned frequently but disinfected irregularly. After infection, geese mostly huddled together, did not move actively, and ate less. Hepatomegaly was observed in the diseased geese. The bacteria purified from the livers of the diseased geese grew as gray-white colonies with a smooth, moist surface and neat edges on the blood agar plate. The diameter of the colonies was 1 mm ± 0.1 mm (Figure 1).

### 3.2. Bacterial Identification

#### 3.2.1. Gram Staining

**Figure 2:**
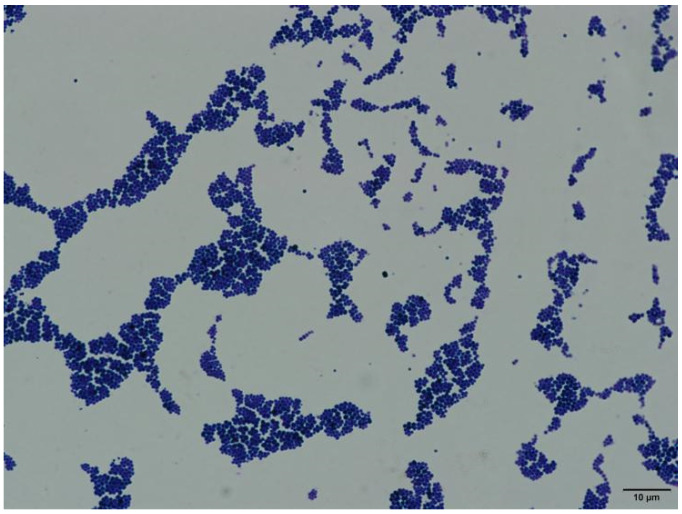
Gram staining of the isolated bacteria (1000× magnification).

Under an oil immersion microscope (1000× magnification), the bacteria appeared as Gram-positive cocci and the cells were small and uniformly ovoid-shaped spheres with diameters ≥ 1.0 µm (Figure 2).

#### 3.2.2. Bacterial Biochemical Test

The bacteria could only ferment glucose, maltose, and sucrose, but not lactose, mannitol, sodium citrate, hydrogen sulfide, or urea (Table 1). The results were consistent with the biochemical characteristics of *Rothia nasimurium*.

#### 3.2.3. MALDI-TOF MS Identification

The bacteria were identified using MALDI-TOF MS. The result showed that the score was 2210 and database searching revealed that the bacterium was *Rothia nasimurium*.

### 3.3. Sequence Analysis of 16S rRNA of the Bacteria

**Figure 3:**
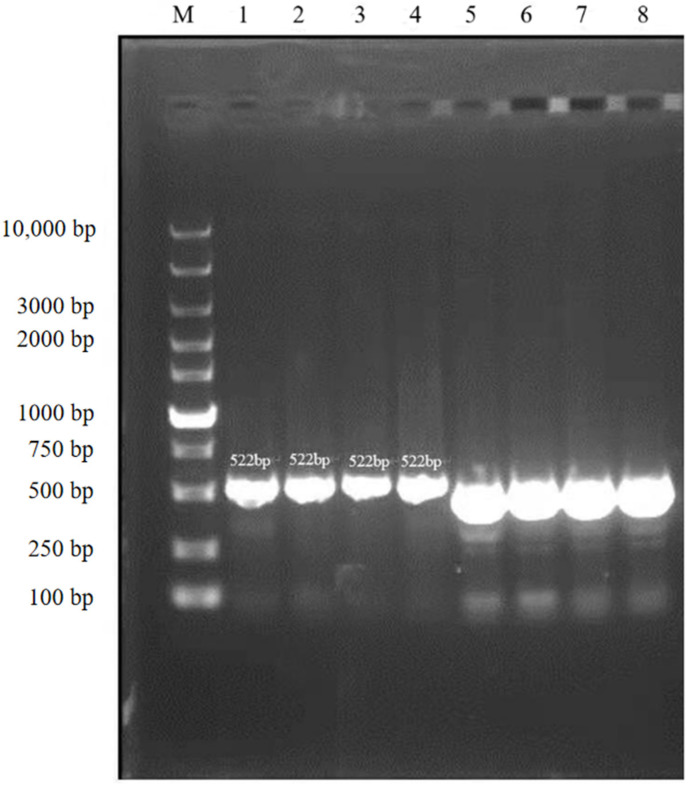
Agarose gel electrophoresis (cropped).

**Figure 4:**
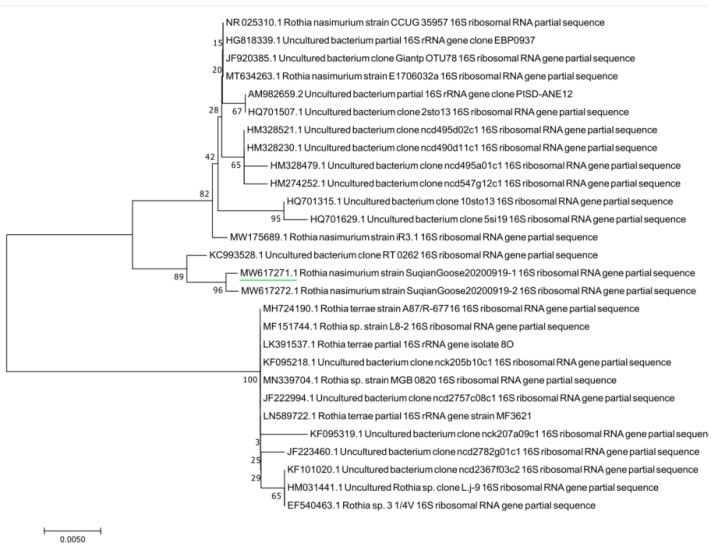
Phylogenetic tree based on bacterial 16S rRNA genes.

The results of agarose gel electrophoresis (Figure 3) showed that the PCR amplicons from the randomly selected pure cultured bacteria were all about 500 bp, which was consistent with the expected fragment size. The phylogenetic tree showed that the tested bacteria clustered to the branch of *Rothia nasimurium* (Figure 4), which agreed with the results of the other identification experiments. The GenBank accession number of the 16S rRNA sequence of this isolate is MW617271.

Lane M: DNA Marker; lanes 1–4: bacterial samples stained with ethidium bromide; lanes 5–8: bacterial samples stained with loading buffer.

### 3.4. Antibiotic Susceptibility Testing

The sensitivities of the *Rothia nasimurium* isolate to different drugs varied (Table 2). Using the CLSI method, the results of the drug sensitivity test showed that the level of the inhibition of amikacin was the highest (diameter = 24 mm), followed by cefazolin, fosfomycin, and ampicillin/sulbactam, which showed similar inhibition levels. However, the isolate was resistant to the other 16 tested drugs, with the diameter of the bacteriostatic rings for most drugs being 0, i.e., these drugs had no significant bacteriostatic effect. Thus, the isolated strain showed multidrug resistance.

According to the results of antibiotic susceptibility testing, amikacin was the most recommended drug for treatment; however, attention should be paid to the usage and dosage to avoid the harm caused by antibiotic abuse.

In addition, we noticed that the results for beta-lactam antibiotics were not in agreement with each other; therefore, the experiment was repeated. However, the results were the same as those of the first experiment.

### 3.5. Animal Regression Experiment

**Figure 5:**
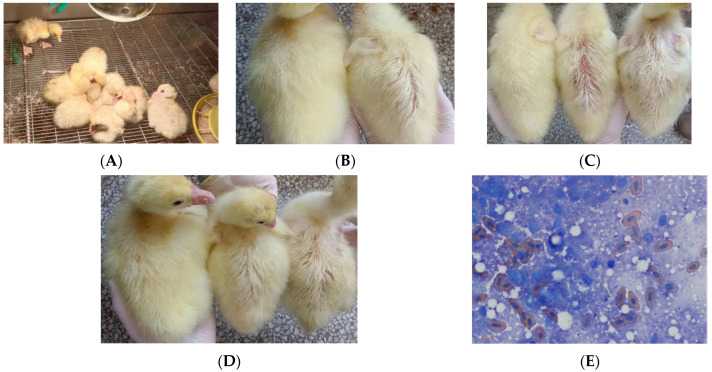
(**A**) Two days post infection. (**B**) Left: hair condition of the control Gosling. Right: hair condition of the intraperitoneal injection Gosling with 5 × 10^7^ cfu. (**C**) Left: hair condition of the control Gosling. Middle: hair condition of the intraperitoneal injection Gosling with 5 × 10^7^ cfu. Right: hair condition of the intraperitoneal injection Gosling with 5 × 10^5^ cfu. (**D**) Left: hair condition of the control Gosling. Middle: hair condition of the perfusion group with 5 × 10^7^ cfu. Right: hair condition of the perfusion group with 5 × 10^5^ cfu. (**E**) Swiss staining of liver tissue (1000 × magnification).

The goslings were observed twice every morning and evening. Before the animal regression experiment, all the goslings were healthy and showed great activity. At 2 days post-infection, there was significant change in their behavior, such as inactivity, crouching, and unsteady gait. They had no desire to move actively, and suffered loss of appetite. All the manifestations were consistent with those of naturally infected geese (Figure 5A). In addition, the intraperitoneal injection group with 5 × 10^7^ cfu showed slight depilation on their backs (Figure 5B). At 3 days post infection, the back depilation of the goslings was significantly aggravated in the intraperitoneal injection group; it was observed that the symptom of the 5 × 10^7^ cfu group was more pronounced than that of the 5 × 10^5^ cfu group (Figure 5C) and slight depilation was also found in the perfusion group (Figure 5D). No significant change was observed in the negative control group, which appeared active and had a good appetite, with no depilation being observed. The positive goslings with obvious symptoms were killed; slight hepatomegaly was also observed and the bacteria in their livers, spleens, and blood were isolated and cultured. The colonies were identified by MALDI-TOF MS and a slice of liver tissue was gently glued to a slide, fixed using heat, subjected to Swiss staining, and then observed under a microscope, which revealed cocci-like bacteria (Figure 5E). The results of MALDI-TOF MS also showed that the isolated bacteria were *Rothia nasimurium*. Thus, *Rothia nasimurium* was successfully isolated from the goslings, which proved that this bacterium could survive in geese and cause them disease.

## 4. Discussion

In previous reports, *Rothia nasimurium* was identified and isolated as part of the normal flora in animals. To date, *Rothia nasimurium* has not received much attention in veterinary microbiology, and some scholars believe that this bacterium is an opportunistic pathogen; however, there is no experimental basis for this claim [6].

In the present study, a strain of *Rothia nasimurium* was identified in diseased geese, which enriches our knowledge of pathogenic microorganism species in geese. The result of the antibiotic susceptibility testing showed that the isolated strain has strong drug resistance, which means it may become a potential threat to public health. The increase of antimicrobial resistance is a global problem for both human and animal health. The goose industry must perform well in terms of environmental disinfection and sterilization [24,25]. At the same time, targeted therapy is also essential because of its higher treatment efficiency [26,27]. Through the antibiotic susceptibility testing, amikacin was identified as the most effective antibiotic against *Rothia nasimurium*. This result provides accurate guidance for clinical antibiotic use, allowing more targeted treatment and reducing the abuse of antibiotics. In addition, the mechanism by cefazolin that acts as the only effective antibiotic in the beta-lactam group requires further investigation. Increased drug resistance of bacteria could be effectively avoided through targeted therapy [28,29,30]. At the same time, the evolution and spread of bacterial drug resistance could be reduced [31].

The result of the animal regression experiment showed that the clinical and pathological signs of the infected goslings were highly similar to those naturally infected geese, and the bacteria could be isolated from the liver and blood again after injection and oral administration. This means that the bacteria can penetrate the body’s immune barrier and pose a health threat, so geese breeders should not ignore the potential harm caused by *Rothia nasimurium*. Furthermore, the serious depilation of the goslings caused by its infection was unexpected and intrigued the researchers. Feathers are very important to poultry, and depilation can cause serious harm to poultry. Therefore, the mechanism of depilation of goslings deserves an in-depth study.

## 5. Conclusions

In this study, a strain of *Rothia nasimurium* was found to be able to survive and cause disease in geese; it could also cause serious depilation of goslings, suggesting that it has potential to harm geese or other animals. Through antibiotic susceptibility testing, we found that the strain showed multidrug resistance, but was most sensitive to amikacin; cefazolin, fosfomycin, and ampicillin/sulbactam were also effective against this bacterium. Considering its multidrug resistance, more attention should be paid to this strain of *Rothia nasimurium* to reduce its potential hazards. The results of the present study could help the goose industry to reduce losses caused by bacterial infection and will help to develop methods for avian endemic control.

## Figures and Tables

**Table 1:** Bacterial biochemical test.

Substrate	Result
Glucose	+
Lactose	−
Maltose	+
Mannitol	−
Sucrose	+
Sodium citrate	−
Hydrogen sulfide	−
Urea	−

**Table 2:** Results of the drug sensitivity test.

Drug Name	Judging Standard	Actual Result	Result	Drug Name	Judging Standard	Actual Result	Result
Tetracycline	≥15, ≤11	0	resistant	Imipenem	≥23, ≤19	16	resistant
Cefepime	≥25, ≤18	0	resistant	Amikacin	≥17, ≤14	24	susceptible
Methoxybenzylaminopyrimidine	≥16, ≤10	0	resistant	Cefoxitin	≥18, ≤14	0	resistant
Cefotaxime/clavulanic acid	≥26, ≤22	19	resistant	Fosfomycin	≥16, ≤12	19	susceptible
Azithromycin	≥13, ≤12	0	resistant	Norfloxacin	≥17, ≤12	0	resistant
Ceftazidime	≥18, ≤14	0	resistant	Ampicillin/sulbactam	≥15, ≤11	19	susceptible
Compound sulfamethoxazole	≥18, ≤13	0	resistant	Aztreonam	≥21, ≤17	0	resistant
Levofloxacin	≥17, ≤13	0	resistant	Chloramphenicol	≥18, ≤12	0	resistant
Cefazolin	≥23, ≤19	25	susceptible	Gentamicin	≥15, ≤12	0	resistant
Meropenem	≥20, ≤15	11	resistant	Azlocillin	≥15, ≤11	0	resistant

Judging standard refers to the CLSI standard, each number is measured in millimeters (mm). Susceptible: actual result higher than the maximum. Resistant: actual result lower than the minimum. Medium: actual result between the maximum and the minimum.

## Data Availability

The datasets used during the current study are available from the corresponding author upon reasonable request. The datasets generated during the current study are available in the GenBank repository [32], (https://www.ncbi.nlm.nih.gov/nuccore/MW617271.1, accessed on 17 February 2021).
